# Correlation Between Serum Ferritin and Hepcidin Levels in Chronic Hepatitis C Patients

**DOI:** 10.7759/cureus.17484

**Published:** 2021-08-27

**Authors:** Maria Gill, Javaria Sharafat, Faiza Ikram, Misbah Ul Qamar, Irum Rehman, Mannal Saleem, Ayesha Noreen, Nadia Khadim, Arusa Horii, Bakhtawar Tahir

**Affiliations:** 1 Physiology, CMH Multan Institute of Medical Sciences, Multan, PAK; 2 Physiology, Akhtar Saeed Medical and Dental College, Lahore, PAK; 3 Physiology, Margalla Institute of Health Sciences, Rawalpindi, PAK; 4 Physiology, Nawaz Sharif Medical College, University of Gujrat, Gujrat, PAK; 5 Physiology, Sargodha Medical College, Sargodha, PAK; 6 Physiology, Lahore Medical and Dental College, Lahore, PAK

**Keywords:** hepcidin, iron, ferritin, hepatitis c, chronic

## Abstract

Background

In addition to the known role of serum ferritin as an inflammatory mediator, its role in the induction of serum hepcidin is yet to be elucidated. This study aimed to identify a correlation between serum ferritin and hepcidin levels in chronic hepatitis C (CHC) patients and healthy individuals.

Methodology

A total of 44 male subjects, selected by convenient sampling technique, were included in this study. The study population was divided into group I including 22 healthy males and group II including age-matched 22 CHC patients. Serum hepcidin and serum ferritin levels of study participants in both groups were assessed. Serum parameters were compared between two groups using the Mann-Whitney U test. Spearman correlation test was applied between serum ferritin and serum hepcidin in each group. P-values of ≤0.05 were considered significant.

Results

The median values of serum ferritin in group I and group II were in the normal range, though serum ferritin of CHC patients was significantly higher than the healthy population (p = 0.03). The median values of serum hepcidin in both groups were below the normal range. In CHC patients, a negative nonsignificant correlation (rho = -0.34, p = 0.13) was observed between serum ferritin and serum hepcidin. A positive nonsignificant correlation (rho = 0.19, p = 0.4) was observed between serum ferritin and serum hepcidin in the healthy population.

Conclusions

Our study could not bring forth any conclusive remarks in favor of serum ferritin as an inflammatory mediator raising serum hepcidin levels among CHC patients. A negative nonsignificant correlation between studied parameters in CHC patients may indicate the involvement of some other factor such as hepatitis C virus in the reduction of serum hepcidin levels.

## Introduction

After the discovery of serological markers for hepatitis A and B from 1970 to 1980, another obnoxious virus was discovered and held accountable for most transfusion-induced hepatitis, that is, non-A, non-B hepatitis (NANBH) [1]. In the late 1980s, serological diagnosis of hepatitis C virus (HCV) was made possible by the successful molecular cloning of the viral genome and, in 1989, NANBH was replaced with hepatitis C [1]. In 2015, the World Health Organization reported an estimated worldwide prevalence of hepatitis C to be 2.3% and 1.5% in eastern Mediterranean and European regions, respectively, with 70 million people affected globally [2]. Known risk factors for hepatitis C transmission are the reuse of syringes, intravenous drug abuse, organ transplantation, and exposure to blood products [3]. Of these risk factors, blood transfusion and dialysis are the leading causes for the transmission of hepatitis C [3].

CD81 and scavenger receptor class B type 1 are known receptors of HCV [4]. After entering the cell via endocytosis, HCV loses its coat, and its positive-sense viral RNA becomes a template for the synthesis of viral proteins and generation of new virus particles, which are further packed and released from host cells via secretory pathway [4]. By altering the membrane permeability, HCV allows calcium to exit from the endoplasmic reticulum (ER) [4]. When calcium enters the mitochondria it inhibits complex I and oxidative phosphorylation. As a result, a cell must depend more on glycolytic pathways for energy. Reactive oxygen species (ROS) are generated which decrease hepcidin expression from hepatocytes [5]. Hepcidin, a 25aa hormone, acts on ferroportin and regulates iron release from enterocytes [6]. Inflammatory conditions, iron stores, and endoplasmic stress upregulate hepcidin. Oxidative stress, anemia, and hypoxia can inhibit hepcidin expression [6]. Decreased hepcidin level increases iron absorption from enterocytes by expressing ferroportin on the basolateral membrane, thus playing a role in iron overload [5]. Oxidative stress due to HCV overrides hepcidin and induces inflammatory signals wherein serum ferritin is an inflammatory marker [7]. Early during chronic hepatitis C (CHC), prominently decreased serum hepcidin levels might be caused by HCV; however, this negative viral impact may be masked by increased iron accumulation in the form of ferritin [7]. Increased iron stores in blood and tissues stimulate hepcidin expression via bone morphogenic proteins (BMPs) [8]. Increased hepcidin expression as a result of iron accumulation may influence the regulation of serum and tissue iron levels via BMPs [8].

This study aimed to determine serum hepcidin and serum ferritin levels in CHC patients. We intended to determine any correlation between serum ferritin and serum hepcidin levels in CHC patients and healthy individuals. This study can be a gateway for further research targeting hepcidin antagonists and hepcidin agonists as treatment strategies in chronic inflammatory diseases, such as chronic liver or kidney diseases, and malignancies, and thus slowing their progression.

## Materials and methods

A total of 44 male subjects selected by convenient sampling technique were included in the study after taking approval from the Institutional Review Board of the University of Health Sciences, Lahore. A total of 44 subjects between the ages of 35 and 65 were selected from the Medical Unit of Jinnah Hospital Lahore. The study population was divided into two groups: group I including 22 healthy males and group II including 22 male CHC patients. Only CHC patients diagnosed by polymerase chain reaction for hepatitis C RNA were included in group II. Patients having any potential factor other than hepatitis C that could affect serum ferritin, for example, blood transfusion/donation and iron supplementation, were excluded from the study. Patients with known superimposed acute or chronic infections and terminally ill CHC patients were excluded during sampling. Females were excluded because commenting on the iron status of females during the menstrual cycle, menorrhagia, and menopause is difficult.

After obtaining informed consent, trained personnel collected about 5 mL of venous blood samples from subjects in both groups under strict aseptic measures. This blood sample was then used to determine serum hepcidin and ferritin levels. As per the instruction manual of enzyme-linked immunosorbent assay (ELISA) kits, serum samples were appropriately stored in carefully labeled Eppendorf Tubes. Serum analysis of hepcidin and ferritin was performed using human ELISA kits manufactured by Glory Science Co., (Del Rio, USA) and Diametra (Spello, Italy), respectively. An automated enzyme immunoassay analyzer (CODA®, Bio-Rad Laboratories, CA, USA) was used.

The data were analyzed using SPSS version 26 (IBM Corp., Armonk, NY, USA). Normality of data was assessed using the Shapiro-Wilk test, considered significant at a p-value of ≤0.05. For descriptive analysis of non-normally distributed quantitative variables, median values along with interquartile range (IQR) were computed. Non-normally distributed serum parameters were compared between the two study groups using the Mann-Whitney U test. To determine the correlation between nonuniformly distributed quantitative variables, the Spearman correlation test was used. All analyses considered a p-value of ≤0.05 as statistically significant.

## Results

**Figure 1:**
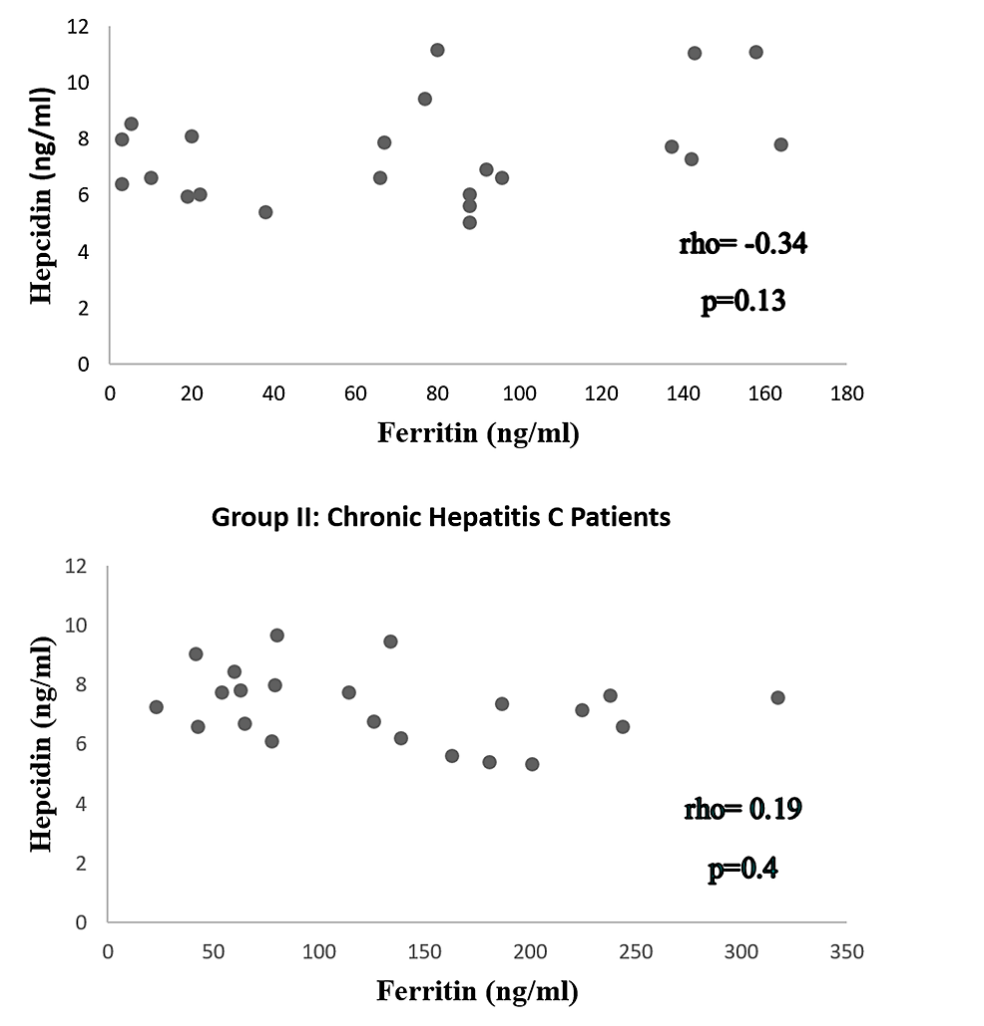
Correlation between hepcidin and ferritin in CHC patients and the healthy population. *Significant at p ≤ 0.05. CHC: chronic hepatitis C

A total of 44 male subjects were included in this study, with a mean age of 50.35 ± 13.28 years. The analysis of dependent variables including serum ferritin and serum hepcidin in both groups revealed that data were non-normally distributed. The median of observed serum ferritin levels of groups I and II fell within the normal range, although median values of serum ferritin of group II were higher compared to group I. Comparison of serum ferritin levels among healthy subjects (group I) and CHC patients (group II) via the Mann-Whitney U test showed significant results (p ≤ 0.03) (Table 1). The median serum hepcidin levels in both groups were below normal. Comparison of the median value of serum hepcidin levels in both groups did not show any significant difference (p = 0.94) (Table 1). There was a positive but nonsignificant correlation between ferritin and iron (rho = 0.19, p = 0.4) in the healthy population (Figure 1). A negative but nonsignificant correlation was observed between ferritin and hepcidin in CHC patients (rho = -0.34, p = 0.13) (Figure 1).

**Table 1:** Comparison of serum ferritin and hepcidin levels among the healthy population and CHC patients. *Significant at p ≤ 0.05. CHC: chronic hepatitis C; IQR: interquartile range

Parameter	Normal range	Group I (Healthy) Median (IQR)	Group II (CHC) Median (IQR)	Mann-Whitney U test	
				U	p-value
Serum ferritin (ng/mL)	20–400	78.5 (3–164)	120 (23–317)	151.5	0.03*
Serum hepcidin (ng/mL)	29–254	7.07 (5.04–11.16)	7.30 (5.33–9.65)	239	0.94

## Discussion

The results of this study showed that serum ferritin levels were significantly higher among CHC patients compared to healthy individuals. In addition, serum hepcidin levels were negatively correlated with serum ferritin levels among CHC patients, in contrast to healthy individuals where serum hepcidin and ferritin were positively correlated.

Raised ferritin levels in CHC patients compared to healthy controls can reflect a dual process, that is, iron overload and ongoing inflammation in CHC infection [9]. According to a review article published in 2017, several studies have variably associated raised serum ferritin levels and hepatic ferritin stores among CHC patients with iron overload or hepatic inflammation [10]. Moreover, the observation of reduced hepcidin levels among CHC patients is consistent with previous literature, suggestive of reduced hepcidin levels causing iron overload among these patients [6,10,11]. Although still debatable, reduced hepcidin levels in CHC patients are theoretically proposed to be caused by oxidative stress-induced epigenetic alteration of hepcidin expression [5]. However, Tsochatzis et al. in 2010 proposed that serum hepcidin tends to increase among CHC patients under the influence of proinflammatory cytokines, specifically interleukin-6 [12].

In group I (healthy population), a nonsignificant positive correlation between serum hepcidin and serum ferritin may be indicative of the downregulation of serum hepcidin because of reduced iron stores [13]. A negative correlation between serum ferritin and hepcidin among CHC patients can be a clue that the HCV might be involved in the downregulation of hepcidin [14]. Consistent with our findings, another study conducted by Aoki et al. on 96 CHC patients at Athens University concluded that iron loading seen in CHC patients was not due to an inappropriate amount of serum hepcidin [15]. This study reported that compared to other inflammatory diseases, serum hepcidin did not correlate with other inflammatory markers, rather it was significantly negatively correlated with serum ferritin levels, as seen in the current study [15].

In contrast, another study conducted in 2010 among 96 CHC patients at Athens University reported a positive correlation of serum hepcidin with aspartate aminotransferase and insulin resistance but no correlation with serum ferritin [12]. Similarly, no significant correlation was found between serum ferritin and hepcidin in another study conducted in 2021 among thalassemia patients in Thailand [16]. However, a significant correlation between serum ferritin and hepatic iron grading was reported by a study conducted among 72 CHC patients [9]. This study suggested assessing the hepatic iron overload in CHC patients, as serum ferritin can be an indirect marker [9].

This study is limited by its relatively smaller sample size. The inclusion of a larger CHC population might have resulted in a significant correlation between serum hepcidin and ferritin. Further studies including a larger sample size should be conducted using hepatic iron grading as a marker of iron overload, in addition to serum ferritin levels. The information thus established can help devise relevant therapeutic approaches to limit the disease progression imposed by iron overload.

## Conclusions

The current study was conducted to explore the correlation between serum hepcidin and ferritin levels among CHC patients. Along with below-normal serum hepcidin levels, this study reported a significantly higher serum ferritin level among CHC patients compared to healthy controls. Moreover, serum hepcidin levels were negatively but insignificantly correlated with serum ferritin levels among CHC patients, in contrast to healthy individuals where serum hepcidin and ferritin were positively correlated. Thus, our study could not conclusively remark in favor of serum ferritin as an inflammatory mediator raising serum hepcidin levels among CHC patients. A negative, nonsignificant correlation between studied parameters in CHC patients may indicate the involvement of other factors such as HCV in the reduction of serum hepcidin levels.
